# Real-life safety of peanut oral immunotherapy: Results from a French multicenter observational study

**DOI:** 10.1016/j.jacig.2025.100628

**Published:** 2025-12-17

**Authors:** Elodie Michaud, Flore Amat, Céline Lambert, Amandine Divaret-Chauveau, Antoine Deschildre, Peanut Oral Immunotherapy Working Party from the French Society of Allergology, A. Nemni, M. Voidey, T. Moraly, P. Bierme, D. Sabouraud, L. Couderc, D. Caimmi, A. Dupré Latour

**Affiliations:** a Paediatric Allergy Department, CHU Clermont-Ferrand, Clermont-Ferrand, France; b Pediatric Pulmonology and Allergology Department, Hopital Robert Debré, Paris, France; c INSERM U1018—Center for Research in Epidemiology and Population Health (CESP), “Integrative Respiratory Epidemiology” Team, Villejuif, France; d Biostatistics Unit, DRCI, CHU Clermont-Ferrand, Clermont-Ferrand, France; e Paediatric Allergy Department, University Hospital of Nancy, Vandoeuvre-les-Nancy, France; f UR 3450 DevaH, Faculty of Medicine, University of Lorraine, Vandoeuvre-les-Nancy, France; g Pediatric Pulmonology and Allergology Department, Pôle enfant, Hôpital Jeanne de Flandre, CHRU de Lille and Université Nord de France, Lille, France

**Keywords:** Peanut, oral immunotherapy, allergic reaction, external cofactors, real-life study, therapeutic education

## Abstract

**Background:**

Peanut oral immunotherapy (POIT) has been widely used in France for more than 10 years. However, the overall “real-life” safety of POIT has not been evaluated to date.

**Objective:**

We sought to describe the number, severity, and circumstances of allergic reactions (ARs) in patients undergoing POIT.

**Methods:**

We performed a retrospective multicenter study from November 2019 to July 2021 in 12 French centers, including patients with peanut allergy who were older than 3 years and treated by POIT for 6 months or more. Data collected from the patients’ charts about ARs occurring during the previous year included the number, severity (using the Astier score grades 1-5), and circumstances of all immediate allergic reactions (IARs) and non-IARs.

**Results:**

Among the 295 patients included, 46 (15.6%) experienced an IAR, accounting for a total of 75 IARs. The IARs were mainly grade 1; however, 22 (29.3%) were defined as a serious systemic reaction (ie, Astier score grade ≥ 3) and 8 (10.6%) were managed by epinephrine injection at home. Proven cofactors were involved in 38 of 73 IARs (52.1%): exercise in 65.8%, fatigue in 18.4%, stress in 13.2%, irregular peanut intake in 5.3%, and pollen exposure in 5.3%. The use of epinephrine was more frequent when a cofactor was involved (0% vs 18.4%; *P* = .01). Among the 279 patients with available data, 32 (11.5%) experienced non-IARs, mainly chronic abdominal pain (20 [62.5%]).

**Conclusions:**

Although POIT is safe for most patients, some severe IARs influenced by cofactors may occur several months after the beginning of the process. These results highlight the overriding importance of maintaining therapeutic education, especially about risk cofactors, throughout oral immunotherapy programs.

The “Etude Longitudinale Française depuis l’Enfance” cohort estimates the prevalence of peanut allergy in France at 0.93% in children up to age 5.5 years.^1^ Peanut allergy very often begins at preschool age and persists into adulthood in about 70% of patients.^2^ It is frequently associated with serious allergic reactions (ARs), which can be lethal.

The first-line treatment for food allergy remains allergen avoidance. Nonetheless, a prolonged elimination diet is difficult: anaphylactic accidents do occur and are unpredictable. Furthermore, the social burden of acute ARs is considerable. Overall, these factors have a negative impact on the quality of life of both the patients and their families.^3^

Oral immunotherapy (OIT) now offers new opportunities in the management of patients with allergy. Studies on peanut oral immunotherapy (POIT) are numerous and globally show a clinical efficacy.^4^, ^5^, ^6^, ^7^ The results of these studies have raised hopes that patients would be protected against severe accidental ARs, even if sustained unresponsiveness after stopping peanut exposure has not yet been clearly demonstrated.^7^

Nevertheless, because of their frequency and potential severity,^6^ ARs remain one of the obstacles to widespread adoption of POIT in clinical settings. However, although the standardized therapy Palforzia has now been validated in the United States^5^ and in a few European countries, it is scarcely used in France where POIT has been routine care for more than 10 years. As a first step toward developing a set of national guidelines on the basis of a validated protocol, the aim of this study was to describe the number, severity, and circumstances of ARs among patients undergoing POIT in a “real-life” context.

## Methods

### Study design

We performed a retrospective multicenter study from November 2019 to July 2021, involving 12 French expert POIT centers: Aulnay-sous-Bois, Besançon, Clermont-Ferrand, Lille (2 centers), Lyon, Nancy, Paris, Reims, Rouen, Montpellier, and Saint-Etienne hospitals. We included patients older than 3 years and treated with POIT as routine care for at least 6 months. All participants had a documented peanut allergy diagnosed by an allergologist defined as a positive skin prick test (SPT) result or specific serum IgE (≥0.35 kUA/L) to peanut together with a positive oral food challenge (OFC) result unless an immediate reaction (within 2 hours of exposure) to peanut had been documented in the previous 18 months. All the patients and their families attended sessions to educate them about POIT, especially about how to manage cofactors.

In the absence of guidelines at the time of the study, exclusion criteria for POIT were at the allergist’s discretion.

### Outcomes

The primary outcome of the study was the occurrence of an immediate allergic reaction (IAR) during the previous year of POIT. Secondary outcomes were the description of the severity of the IARs, the circumstances of the IARs, and the occurrence of non-IARs.

### Data collection

Data were obtained from the patients’ charts. We collected data related to age, sex, allergic comorbidities (ie, active atopic dermatitis, asthma, rhinitis, and other food allergies), and peanut allergy before POIT (ie, history of anaphylaxis before POIT and cumulative eliciting dose). The cumulative eliciting dose referred to either the total ingested dose until a reaction occurred during the OFC or the total ingested dose estimated by the allergist for patients reporting ARs within the previous 18 months. Data regarding the targeted dose, current dose of POIT, time from the beginning of POIT, duration from initial food challenge to the beginning of POIT, the diameter of the SPT wheals, and peanut-specific IgE levels before and during POIT were also collected. The number, severity, and circumstances of all recorded IARs during the previous year of POIT were collected for each patient. We defined an IAR as any AR appearing within 6 hours following peanut intake, and these IARs were classified using the Astier score adapted from the Ewan and Clark scale^8^^,^^9^: grade 1, abdominal pain that resolved without requiring medical treatment, rhinoconjunctivitis, urticaria with fewer than 10 papules, and rash (eczema onset); grade 2, 1 organ involved, abdominal pain requiring treatment, generalized urticaria, nonlaryngeal angioedema, mild asthma (cough or <20% fall in peak expiratory flow); grade 3, 2 organs involved; grade 4, 3 organs involved or asthma requiring treatment or laryngeal edema or hypotension; and grade 5, cardiac and respiratory symptoms requiring hospitalization in intensive care. Serious systemic reaction was defined as a grade 3 or higher.^10^ The symptoms of the IARs and their severity were double-checked by 2 independent authors (F.A. and E.M.) to ensure appropriate classification.

We also recorded the way each IAR was managed: use of epinephrine, a call to the allergist, emergency room visit, and need for hospitalization. To collect the circumstances of the IARs, the clinicians were asked to complete a systematic checklist including stress, physical activity within 2 hours before or after the intake, viral illness, uncontrolled asthma, alcohol intake, use of concomitant medications (eg, steroidal anti-inflammatory drugs or proton pump inhibitors), fatigue, unusual schedule, empty stomach, menstruation, pollen season, and irregular peanut intakes. The clinicians could also provide any other specific details about the IAR if not on the list. The phase of POIT during which the IAR occurred was recorded: the buildup phase corresponding to an increase in peanut intake, mostly on a monthly schedule according to French clinical practice; the maintenance phase corresponding to a fixed daily intake of peanut; and the spacing phase when a slight reduction in frequency is allowed (in practice, the minimum frequency of peanut intake is 3 times a week).

We also analyzed non-IARs throughout the POIT, such as loss of atopic disease control (asthma and atopic dermatitis), eosinophil esophagitis, and recurrent abdominal pain. More precisely, loss of asthma control was taken as being a resurgence of chronic asthma symptoms, such as daytime symptoms, nocturnal symptoms, need for reliever treatment more than twice a week, or limitation in activities.

The number of patients who dropped out because of ARs was also recorded.

### Statistical analysis

This cross-sectional study, conducted on a sample, aimed to generalize the results to the entire target population. To determine the required sample size, a margin of error was defined on the basis of the estimated proportion of the primary outcome. Given an expected proportion of IARs of about 50% (proportion that requires the largest sample size), at least 267 patients would be needed to achieve a precision of ±6%.

Statistical analyses were performed using Stata software (version 15; StataCorp, College Station, Tex). All tests were 2-sided, with an alpha level set at 5%. Categorical data are presented as numbers and associated percentages and continuous data as mean ± SD or median (25th; 75th percentiles), depending on the statistical distribution. The proportion of patients who experienced at least 1 IAR is presented with a 95% CI estimated by a binomial distribution. The IARs were compared according to the presence or absence of cofactors using mixed models to take into account the repeated measures per patient (1-5 IARs per patient). Factors associated with having at least 1 IAR or with having at least 1 grade 3 or higher IAR (1 data point per patient) were studied using the chi-square test or the Fisher exact test for categorical variables and the Student *t* test or the Mann-Whitney test for continuous variables. Spearman rho coefficient was used to assess correlations.

### Ethics

This retrospective, noninterventional study based on data from medical charts was approved by the ethics committee of the coordinating center (Clermont-Ferrand University Hospital) under the reference 2019/CE 48. Data were anonymized according to good clinical practice, and the study complied with French “Commission Nationale Informatique et Libertés” recommendations.

## Results

A total of 295 patients with peanut allergy were included. They were predominantly boys (63.1%), with an average age of 10.8 ± 4.3 years (range, 2-34 years). About one-third were older than 12 years. Most of the patients had multiple atopic comorbidities. Patients were highly sensitized to peanut (median SPT wheal, 10 mm [7; 14]; median peanut-specific IgE level, 44 kUA/L [7; 100]; median recombinant Ara h 2–specific IgE level, 26 kUA/L [4; 74]), and 36.8% (99 of 269) of them had experienced anaphylaxis due to peanut before POIT. The median cumulative eliciting dose during the AR before POIT was 54 mg of peanut proteins (26; 179) (Table I). The duration between the last OFC and the initiation of POIT was usually less than 6 months. The median targeted dose of peanut proteins for POIT was 500 mg (300; 600). The median current dose of peanut proteins was 300 mg (75; 500), resulting in a significant increase (*P* < .001) from the beginning of POIT (initial tolerated dose, 38 mg of peanut proteins [13; 100]; initial intake at home, 4 mg [2.5; 17.5]). Higher current doses were observed in patients older than 12 years (363 mg of peanut proteins [125; 600]) compared with younger patients (250 [63; 500]; *P* = .02), with a significant although low correlation between dose and age (Spearman rho coefficient, 0.24; *P* < .001).

**Table I:** Demographic characteristics and clinical presentation of patients diagnosed with IgE-mediated peanut allergy

Characteristics	Total (N = 295)
Demographic characteristics	
Age (y)	10.8 ± 4.3
Age >12 y	96 (32.5)
Sex: male	186 (63.1)
Active atopic dermatitis	55 of 280 (19.6)
Recurrent wheezing/asthma	103 of 281 (36.7)
Allergic rhinitis	120 of 281 (42.7)
Other food allergies	132 (44.7)
Characteristics of peanut allergy	
SPT wheal (mm) (n = 225)	10 (7; 14)
Peanut-specific IgE level (kUA/L) (n = 239)	44 (7; 100)
Ara h 2–specific IgE level (kUA/L) (n = 278)	26 (4; 74)
Anaphylaxis history before POIT	99 of 269 (36.8)
Cumulative eliciting dose during AR (mg of protein) (n = 286)	54 (26; 179)

Data are presented as n (%), mean ± SD, or median (25th; 75th percentiles). In the first column, the number of available data is presented in parentheses when there are missing data.

Among the 12 centers, 7 (accounting for 40.0% of patients) followed a monthly updosing schedule, 4 (accounting for 33.6% of patients) a biweekly schedule, and 1 (accounting for 26.4% of patients) a weekly schedule.

At the time of evaluation, 13 patients (4.4%) had discontinued the POIT. Among the remaining 282 patients, 126 (44.7%) were in the buildup phase, 136 (48.2%) in the maintenance phase for 25 months (12; 42), and 18 (9.9%) in the spacing phase for 8 months (6; 12). These data were missing for 2 patients. The median duration of the POIT was 26 months (13; 51), and the length of time before reaching the maintenance dose was about 9 months (6; 12).

### Summary of IARs

Among the 295 patients, 46 (15.6%; 95% CI, 11.6%-20.2%) had experienced at least 1 IAR in the previous year, accounting for 75 IARs. Of these 46 patients, 26 (56.5%) had 1 IAR, 15 (32.6%) had 2, 3 (6.5%) had 3, and 2 (4.3%) had 5. The IARs mainly occurred during the buildup phase (54 [72.0%]). Most of the IARs (70 [93.3%]) occurred at home. They were mostly grade 1 (38 [50.7%]) or grade 2 (15 [20.0%]). The median trigger dose was 125 mg (36; 300) of peanut proteins. External cofactors were identified in 38 of 73 IARs (52.1%). The identified cofactors in these 38 IARs were as follows: physical activity in 25 IARs (65.8%), fatigue/sleep deprivation in 7 (18.4%), stress in 5 (13.2%), irregular peanut intake in 2 (5.3%), associated pollinosis in 2 (5.3%), and another context in 3 (7.9%) (cheilitis for 1 and associated unspecified factor for 2). The frequency of external cofactors was higher in the maintenance and spacing phases (70.0% vs 45.3%), although not significant (*P* = .07). Place of reaction, median trigger dose, and age were not significantly different according to the presence or absence of cofactors (Table II).

**Table II:** Details of total IARs and with or without cofactors

Variable	Total (N = 73∗)	Without cofactor (n = 35)	With cofactor (n = 38)	*P* value
Grade of reaction				<.001
1	37 (50.7)	29 (82.9)	8 (21.1)	
2	15 (20.5)	5 (14.3)	10 (26.3)	
3	13 (17.8)	1 (2.9)	12 (31.6)	
4	8 (11.0)	0 (0.0)	8 (21.1)	
Phases of POIT				.07
Buildup phase	53 (72.6)	29 (82.9)	24 (63.2)	
Maintenance and spacing phases	20 (27.4)	6 (17.1)	14 (36.8)	
Reaction at home	5 (6.8)	3 (8.6)	2 (5.3)	.58
Epinephrine use	7 (9.6)	0 (0.0)	7 (18.4)	.01
Other medications used				
Antihistamines	37 (50.7)	15 (42.9)	22 (57.9)	.34
Systemic corticosteroids	21 (28.8)	1 (2.9)	20 (52.6)	<.001
Beta2mimetics	18 (24.7)	3 (8.6)	15 (39.5)	.02
Eliciting dose (mg of protein) (n = 70)	125 (36; 300)	73 (27; 250)	300 (90; 450)	.22
Age (y)	11.2 ± 4.1	10.3 ± 3.4	11.9 ± 4.6	.16
Age >12 y	20 (27.4)	7 (20.0)	13 (34.2)	.30

Data are presented as n (%), mean ± SD, or median (25th; 75th percentiles). In the first column, the number of available data is presented in parentheses when there are missing data.

∗ The number of IARs is 73 instead of 75 because information on the presence or absence of a cofactor was missing for 2 reactions.

No significant association was found between having at least 1 IAR and peanut-specific IgE levels (42 kUA/L [5; 100] in the group without any IAR vs 55 kUA/L [18; 100] in the group with at least 1 IAR; *P* = .11), nor with recombinant Ara h 2–specific IgE levels (26 kUA/L [3; 77] vs 31 kUA/L [11; 69]; *P* = .38). None of the following factors were significantly associated with having at least 1 IAR: sex (*P* = .32), age (*P* = .69), any atopic comorbidity (*P* = .28, .55, and .80 for atopic dermatitis, asthma, and rhinitis, respectively), duration of POIT (*P* = .06), cumulative eliciting dose (*P* = .79), targeted dose (*P* = .35), and achieved dose (*P* = .10) (data not shown). However, the updosing schedule was significantly associated with having at least 1 IAR, with 56.5% of patients following a biweekly schedule, 28.3% of patients following a monthly schedule, and 15.2% of patients following a weekly schedule (*P* = .001).

### IAR management

Data related to the management of the IARs were available for 71 IARs: most (61 [85.9%]) were self-managed without seeking medical advice; 2 (2.8%) were managed after a call to the allergist for medical advice; 8 (11.3%) resulted in an emergency room visit, with 1 (1.4%) requiring hospitalization overnight. The medications used to manage the IARs are provided in Table II. Twenty-six IARs (34.7%) self-resolved.

### Serious systemic reactions and use of epinephrine

Twenty-two (29.3%) serious systemic reactions (ie, Astier score grade ≥ 3) occurred in 15 patients (5.4%). No grade 5 reactions were recorded.

Peanut-specific IgE levels were significantly higher in the group with at least 1 grade 3 or higher IAR compared with the group without (100 kUA/L [54; 181] vs 41 kUA/L [5; 100]; *P* = .003). Recombinant Ara h 2–specific IgE levels were also higher in the group with at least 1 grade 3 or higher IAR (43 kUA/L [17; 90] vs 22 kUA/L [4; 73]), although it did not reach significance (*P* = .06). None of the following factors were significantly associated with having at least 1 grade 3 or higher IAR: sex (*P* = .40), age (*P* = .24), any atopic comorbidity (*P* = .74, .62, and .75 for atopic dermatitis, asthma, and rhinitis, respectively), history of anaphylaxis (*P* = .42), targeted dose (*P* = .13), updosing schedule (*P* = .37), and achieved dose (*P* = .72) (data not shown).

Epinephrine was used for 8 of the 75 IARs (10.7%), including 6 of the 22 serious systemic reactions (27.3%). Epinephrine was used mostly during the buildup phase (7 of 54 [13.0%]), with 1 use only during the maintenance and spacing phases (1 of 21 [4.8%]); however, this was not statistically significant.

**Fig 1:**
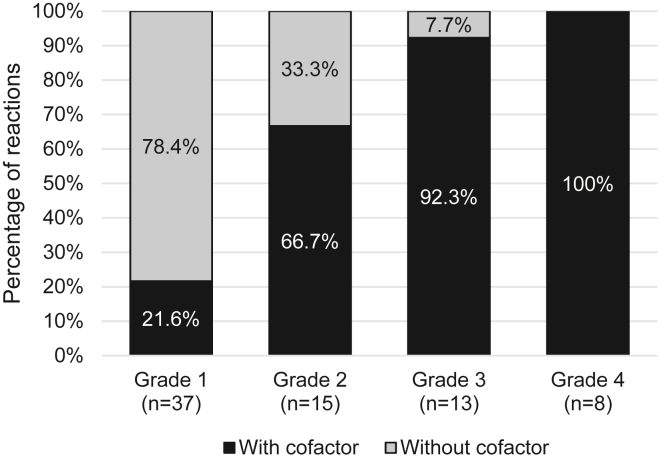
Presence of cofactors according to the grade of reactions.

The frequency of an identified cofactor increased along with the severity of the IAR (Fig 1).

The use of epinephrine also increased when an identified cofactor was present (18.4% vs 0%; *P* = .01), such as the use of other medications (Table II). No significant difference in the frequency of the use of epinephrine was identified according to any atopic comorbidity (*P* = 1.00, .99, and .06 for atopic dermatitis, asthma, and rhinitis, respectively), cumulative eliciting dose (*P* = .25), place of reaction (*P* = 1.00), or age (*P* = .63).

### Non-IARs

Among the 279 patients with available data, 32 (11.5%) experienced non-IARs. Of these 32 patients, 20 (62.5%) complained about chronic abdominal pain, 4 (12.5%) experienced a loss of asthma control, and 4 (12.5%) experienced a loss of atopic dermatitis control. No cases of eosinophilic esophagitis were recorded.

Finally, 111 of the 291 patients (38.1%) presented a peanut aversion.

### POIT dropout cases

Thirteen patients discontinued POIT for the following reasons: excessive treatment-related constraints (7 [53.8%]), taste aversion (7 [53.8%]), and excessive and/or recurrent gastrointestinal symptoms (2 [15.4%]). Other causes (6 [46.2%]) for discontinuation were anxiety, chronic urticaria, loss of asthma control, and lack of compliance. Dropouts occurred during the buildup phase.

## Discussion

In this real-life study describing POIT in a large population of patients in France, most of the patients (84.4%) did not experience any IARs. This corroborates that POIT is generally well tolerated as has already been described in the PALISADE and ARTEMIS research cohorts^4^^,^^5^^,^^11^^,^^12^ and in a 2020 retrospective real-life study.^13^ However, a 2019 meta-analysis (including 12 randomized controlled trials) showed that OIT versus no OIT significantly increased anaphylaxis risk (risk ratio, 3.12; 95% CI, 1.76-5.55), with a high degree of certainty and low heterogeneity, similarly during the buildup and maintenance phases.^6^ In another systematic review and meta-analysis conducted in 2022, de Silva et al^14^ concluded that although some selected patients may benefit from POIT, there are also adverse reactions albeit mainly mild.

In the present real-life study, we found that IARs were more frequent during the buildup phase, during which more serious systemic reactions were observed. The total number of reactions decreased with phase of immunotherapy, as reported by other authors.^6^^,^^15^^,^^16^ However, the risk persists even after the buildup phase.^17^ de Silva et al^14^ did not find a clear relationship between the total duration of treatment (buildup and maintenance phases) and adverse events or severe adverse events.^14^ However, some of these were reported as occurring after 40 or 60 weeks of treatment. We concur with these findings and underline the importance of providing advice and support throughout POIT follow-up.

Peanut-specific and recombinant Ara h 2–specific IgE levels were higher in the group experiencing a grade 3 or higher IAR compared with the group without. In a recent study, Arnau-Soler et al^18^ showed that levels of peanut-specific and Ara h 2–specific IgE levels were significantly lower in complete responders compared with incomplete responders to POIT. This difference was already present before OIT, suggesting that higher peanut-specific IgE levels may reduce OIT responsiveness. We could hypothesize that POIT is more difficult to perform in patients with higher peanut-specific and recombinant Ara h 2–specific IgE levels.

The risk of epinephrine use is known to be increased during POIT, as confirmed in a recent systematic review from Lodge et al^19^ including 8 randomized controlled trials (relative risk, 2.96; 95% CI, 1.63-5.35, with low risk of heterogeneity bias). In our study, only 27.3% of the severe systemic reactions resulted in the use of an epinephrine autoinjector. In a study by Virkud et al,^17^ 38% of patients with a severe reaction were not managed by an epinephrine injection, especially for reactions at home. Our study highlights once again the underuse of this life-saving treatment, which is linked to several factors (unaware of the treatment, apprehension of using the epinephrine autoinjector by the family and medical professionals, etc). This reinforces the necessity of continuing to deliver clear information to families about the possible requirement of epinephrine during POIT and about the indications and use of epinephrine.

Surprisingly, a faster updosing schedule was associated with the lowest frequency of IARs, although this did not hold for serious systemic reactions. One explanation could be that these patients benefited from more frequent educational input compared with patients following other schedules. Our analysis of the circumstances of the IARs demonstrated a link between the presence of external factors and the occurrence of a serious systemic reaction despite the educational measures delivered before starting POIT. Cofactors have been shown to increase the risk of severe ARs in 58% of cases of food anaphylaxis.^20^, ^21^, ^22^, ^23^, ^24^, ^25^, ^26^, ^27^ Exercise, sleep deprivation, acute infection, psychological factors, extreme air temperatures, and symptoms of pollinosis have all been previously described as contributors not only decreasing the threshold of reactivity but also increasing the risk of severe reactions, especially for exercise and sleep deprivation.^23^ Anagnostou et al^28^ underlined a link between unexpected ARs during food OIT after ingestion of a previously tolerated dose and the presence of a cofactor (exercise, fatigue, exposure to inhalant allergens, infection, or menstruation). In our study, this phenomenon persisted over the course of the POIT. Long-term support through regular monitoring and education within a trusting and solid physician-patient relationship is thus essential to prevent poor adherence and manage situations, which could potentially decrease the usual tolerated dose. Clinicians performing POIT and the families should be aware of the persistent risk of IARs even after desensitization during the maintenance or spacing phase.

Finally, non–IgE-mediated, so-called chronic, side effects are frequent during immunotherapy (1 in 10 patients) and are a reason for discontinuation. In our study, abdominal pain and taste aversion were the most frequently reported symptoms, which is consistent with the literature.^12^ Surprisingly, we did not observe any cases of eosinophilic esophagitis, but this might be due to misdiagnosis because eosinophilic esophagitis may be reported as chronic abdominal pain. In total, 4.4% of the patients in our study discontinued their POIT, which is a little lower than the long-term pooled safety data reported by Bird et al.^29^ The reasons for discontinuation in our study were mainly excessive treatment-related constraints and taste aversion, but anxiety was also reported. In a cohort of 50 families undergoing OIT, Polloni et al^30^ showed that 66% of the patients asked for psychological support for the initial phase (eg, OFC and first maintenance doses), 20% during the updosing phase, 8% during the maintenance phase, and 6% after discontinuation. This highlights the importance of involving families and patients in the medical decision process both before and during POIT, taking families’ constraints and psychological aspects of OIT into account as best as possible.

### Strengths and limitations

This is the first real-life retrospective study to describe POIT ARs in a large population of patients in France. All the participating expert centers provided the patients and their families with detailed therapeutic education and performed regular monitoring with a view to limiting the occurrence of ARs. However, because of the retrospective nature of the study, some ARs may be underestimated. More specifically, assessment of eosinophilic esophagitis was not standardized. The circumstances of the IARs (especially viral infections that are not always detectable) may also not have been exhaustively reported. To avoid the risk of recall bias, future studies should use prospective symptom diaries or electronic tools to log reactions and cofactors. Biological data after initiation of POIT were not always available. Reasons for the underuse of epinephrine should be further explored. Moreover, these results are an overall evaluation of POIT but not of a standardized protocol. POIT practices may differ from one center to another, and some practices may result in a higher risk of ARs. Finally, our data only span a period of 20 months. Running a real-life evaluation after implementation of a standardized protocol encompassing a longer time frame and including quality-of-life assessment, monitoring of compliance, and a matched comparator group would help to contextualize reaction rates and to support risk-benefit assessments.

### Conclusion

Anaphylactic risk persists several months after POIT initiation. Clinicians should not forget that OIT is not a harmless procedure but rather requires careful assessment to minimize the risk of adverse effects, especially severe adverse effects. Because cofactors appear to be crucial in the occurrence of severe reactions, ongoing therapeutic education with experienced practitioners should be a cornerstone of patient follow-up. POIT should be discontinued if not tolerated.

In concrete terms, the clinician should systematically check at each visit that their patient’s expectancies have not changed, continue to educate the patient about how to recognize cofactors and manage anaphylaxis, monitor dietary intake, and adapt protocols for high-risk patients.

## Disclosure statement

Disclosure of potential conflict of interest: F. Amat received consulting fees from AImmune Therapeutics and Stallergenes Greer. E. Michaud received consulting fees from AImmune Therapeutics. A. Divaret-Chauveau received consulting fees from AImmune Therapeutics, ALK, Novartis, and Stallergenes Greer. A. Deschildre received consulting and lecture fees from AImmune Therapeutics, Stallergenes Greer, ALK, and Novartis. D. Caimmi received consulting fees from AImmune Therapeutics and Stallergenes Greer. The rest of the authors declare that they have no relevant conflicts of interest.
